# PhosTryp: a phosphorylation site predictor specific for parasitic protozoa of the family trypanosomatidae

**DOI:** 10.1186/1471-2164-12-614

**Published:** 2011-12-19

**Authors:** Antonio Palmeri, Pier Federico Gherardini, Polina Tsigankov, Gabriele Ausiello, Gerald F Späth, Dan Zilberstein, Manuela Helmer-Citterich

**Affiliations:** 1 Centre for Molecular Bioinformatics, Department of Biology, University of Rome Tor Vergata, Via della Ricerca Scientifica, Rome; 2 Faculty of Biology, Technion-Israel Institute of Technology, Haifa 32000, Israel; 3 Institut Pasteur, CNRS URA 2581, Unité de Parasitologie moléculaire et Signalisation, 75015 Paris, France

## Abstract

**Background:**

Protein phosphorylation modulates protein function in organisms at all levels of complexity. Parasites of the *Leishmania *genus undergo various developmental transitions in their life cycle triggered by changes in the environment. The molecular mechanisms that these organisms use to process and integrate these external cues are largely unknown. However *Leishmania *lacks transcription factors, therefore most regulatory processes may occur at a post-translational level and phosphorylation has recently been demonstrated to be an important player in this process. Experimental identification of phosphorylation sites is a time-consuming task. Moreover some sites could be missed due to the highly dynamic nature of this process or to difficulties in phospho-peptide enrichment.

**Results:**

Here we present PhosTryp, a phosphorylation site predictor specific for trypansomatids. This method uses an SVM-based approach and has been trained with recent *Leishmania *phosphosproteomics data. PhosTryp achieved a 17% improvement in prediction performance compared with Netphos, a non organism-specific predictor. The analysis of the peptides correctly predicted by our method but missed by Netphos demonstrates that PhosTryp captures *Leishmania*-specific phosphorylation features. More specifically our results show that *Leishmania *kinases have sequence specificities which are different from their counterparts in higher eukaryotes. Consequently we were able to propose two possible *Leishmania*-specific phosphorylation motifs.

We further demonstrate that this improvement in performance extends to the related trypanosomatids *Trypanosoma brucei *and *Trypanosoma cruzi*. Finally, in order to maximize the usefulness of PhosTryp, we trained a predictor combining all the peptides from *L. infantum, T. brucei and T. cruzi*.

**Conclusions:**

Our work demonstrates that training on organism-specific data results in an improvement that extends to related species. PhosTryp is freely available at http://phostryp.bio.uniroma2.it

## Background

Protein phosphorylation is the most abundant post-translational modification in both prokaryotic and eukaryotic organisms. This process is regulated through the enzymatic activities of protein kinases and phosphatases. Phosphorylation occurs predominantly on serine, threonine, and tyrosine residues, and has been shown to be a key regulatory switch in a variety of cellular processes, ranging from cell cycle and differentiation to motility and learning [1,2]. In particular *Leishmania *lacks transcription factors and phosphorylation has been proposed as an important regulatory mechanism [3].

Recent advances in mass spectrometry enabled the identification of a large number of phosphorylation sites in most eukaryotes (see [4,5] for a review). Information on the phosphoproteome of parasitic protozoa is also starting to be available. In-depth analyses of the phosphoproteome of parasitic protozoa has only recently been initiated in African Trypanosomes and *Leishmania *[6-10].

These studies reported phosphorylation sites whose sequence did not match known kinase recognition motifs, e.g. 25% of the sites identified by Nett *et al*. [6] were not recognized by either Scansite [11] or Netphos [12]. Moreover the data reveal the presence of phosphorylation events not conserved in orthologous proteins. For instance Hem *et al*. [7] showed that a number of chaperones and heat-shock proteins which are very conserved from *Leishmania *to human possess parasite-specific phosphorylation sites.

These findings implicate that new and more family- or *genera*-specific prediction tools are required. Here we use the results of phosphoproteomic experiments in *Leishmania *to develop a novel method that improves P-site prediction in *Leishmania *and other organisms of the trypanosomatidae group.

The complete spectrum of protein phosphorylation is difficult to assess due to the low stoichiometry of many phosphorylation events and the highly dynamic nature of this modification. Thus the bioinformatic identification of putative phosphorylation sites and the subsequent analysis of these sites by biochemical assays may be an important alternative strategy to discover new phosphorylation events.

Phosphorylation sites prediction tools are usually grouped into two categories: generic and kinase-specific. The first category of prediction tools indicates the phosphorylation state of the site, without making any assumption about the protein kinase responsible for the phosphorylation. Methods in the latter category aim to infer which kinase family is responsible for the phosphorylation event. This information is extremely useful for the elucidation of signaling networks, however experimental data linking a protein kinase to its substrate is available only for a limited number of sites [13,14].

Netphos [12] was the first predictor to use neural networks in 1999, outperforming phosphorylation site identification based on sequence motifs alone. Besides the primary sequence, the structural context is also important in determining whether a site is phosphorylated or not [15,16]. Indeed several predictors such as DISPHOS [17] and PHOSIDA [18] include the predicted structural characteristics of the putative phosphorylation sites.

Protein kinase-specific predictors include NetphosK [19], Scansite [11], KinasePhos [20], PredPhospho [21], GPS [22], pkaPS [23] and PrediKin [24]. NetphosK is the extension of the method Netphos to kinase-specific predictions. Scansite uses Position Specific Scoring Matrices (PSSMs) for 62 different kinase phosphorylation motifs. KinasePhos and PredPhospho use HMMER profiles and Support Vector Machines (SVM) respectively. In both cases the prediction models are trained on sets of peptides phosphorylated by protein kinases of the same family. GPS performs profile searches with short motifs instead of using a machine learning approach. In order to achieve a higher coverage of known phosphorylation sites, the algorithm reduces the strength of the profiles, thus increasing the false positive predictions. PkaPS has been developed to predict protein kinase A-specific phosphorylation sites, based on an extensive analysis of the PKA motifs, thus achieving the best results for these particular predictions. PrediKin is based on the analysis of the contact positions between kinases and substrates in proteins of known structure. The authors were able to associate the identification of specific kinase residues with a corresponding preference in the sequence of the substrate.

Moreover a number of organism-specific prediction systems have been developed in recent years [25-28]. These methods aim at increasing the prediction accuracy by training on peptides derived from single organisms. This approach makes it possible to capture organism-specific differences in known phosphorylation motifs and to reduce false positives arising from kinase families that are under-represented in the organism of interest. Following this line of reasoning, the aim of this work is to use *Leishmania *phosphoproteomics data to develop a tool that improves phosphorylation site prediction in trypanosomatids.

## Results and discussion

### SVM features

The dataset used in this work consists of 1176 phosphorylation sites (966 on serine and 210 on threonine) mapping to 482 phosphoproteins. The sites were identified by mass spectrometry after enrichment on a titanium dioxide column. A portion of this data has already been published [7].

PhosTryp uses an SVM-based approach to predict phosphorylation sites; it was therefore necessary to choose a number of features that describe the sites and were used as inputs to the predictor. The features we included in the SVM are:

∙ the sequence of the peptide

∙ a residue composition feature

∙ the secondary structure and disorder predictions for the site.

The sequence of the peptide is clearly the most important characteristic as shown in previous works [18,29]. We considered a window of +/- 5 positions around the phosphorylation site. An important point is how the sequence is encoded, i.e. transformed in variables that can be used as input to the SVM. We tried two different encodings. The first one was the standard orthogonal binary encoding that essentially considers each position as a collection of 20, mutually exclusive, binary variables, each one representing the presence of a specific amino acid in that position. We also used a different encoding based on the values in a substitution matrix (similar to the one used in [30]). This encoding should better represent the fact that a substitution in a position of the peptide could have little influence on the probability of phosphorylation if the residues have similar physicochemical properties.

Moreover we reasoned that in some cases residues close to the phosphorylation site might have an effect independent of their position. To this end we included a feature that depends on the enrichment of each residue in a +/-2 window around phosphorylation sites with respect to non-phosphorylated serine and threonine residues.

Besides these sequence-dependent features we also included two descriptors of the structural context of the site. Indeed phosphorylation sites are usually located in regions of the protein which are flexible and exposed to the solvent in order to facilitate the interaction with protein kinases [15]. The analysis of our dataset confirmed that phosphorylation sites have a preference for disordered regions and segments of the proteins that have a coiled structure. Indeed 968 (83%) of the positive sites lie in a region predicted as coil compared with 780 (66%) of the negatives. The preference for disorderd regions is also apparent: 521 (44%) of the positives are predicted to be disorderd compared with 362 (30%) of the negatives. The significance of these values was confirmed by performing a Chi-square test on the two contingency tables which yielded a p-value < 2e-16 for coil preference and a p-value < 8e-12 for disorder preference. Therefore we added two binary variables describing whether the sites lie in a disordered region or in a coil.

### Training and testing the SVM

As described in the methods we experimented with various combinations of features, building 4 different SVMs. We used 80% of data as training and 20% as test. Both the positive and the negative peptides in the training set were clustered at the 50% sequence identity level to reduce the redundancy. Moreover we removed the peptides in the test set that had more than 50% identity with one of the peptides used for training. The training data was used to optimize each SVM by performing a 10-fold cross validation for each combination of the gamma, cost and epsilon parameters. The results for each SVM are displayed in table 1.

**Table 1:** Results obtained with four different SVMs with different sequence encoding and features

Sequence encoding	Features	AUC Training	AUC Test non-red
binary	all	0,714 ± 0,060	0,719 ± 0,006

binary	sequence only	0,729 ± 0,039	0,706 ± 0,007

PAM30	all	0,729 ± 0,051	0,737 ± 0,007

PAM30	sequence only	0,724 ± 0,021	0,724 ± 0,007

The SVM using only the sequence in binary encoding and the one using the PAM30 encoding and including all the features achieved the same performance on the training set (AUC = 0.73). However the results on the test set indicate that the latter has a superior performance (AUC = 0.74 ± 0.01) and therefore was used throughout the work. However all the SVMs reached essentially comparable performance levels. The final score threshold used for the prediction is 0.54 and was chosen as the one that maximizes the MCC.

### Comparison with Netphos and NetphosK

We compared PhosTryp with Netphos, that provides generic predictions, and with NetphosK that returns a score for each kinase family, according to the likelihood that kinases from that family are responsible for the phosphorylation. Since NetphosK predictions are kinase-specific, we considered as positive predictions the sites that are predicted to be phosphorylated by at least one kinase family.

We tested Netphos and NetphosK on the same non-redundant test set used for PhosTryp, obtaining an AUC 0.57 ± 0.01 for both methods (see table 2). The performance of these programs is therefore markedly inferior to the one obtained by PhosTryp (0.74 ± 0.01). These values represent the average and standard error of 100 bootstrap replicates (see Methods) and therefore give a reliable picture of the performance differences between the three methods.

**Table 2:** Performance of Nepthos and NetphosK on the *Leishmania *dataset

Method	AUC Test non-red
Netphos	0,569 ± 0,008

NetphosK	0,572 ± 0,008

**Figure 1:**
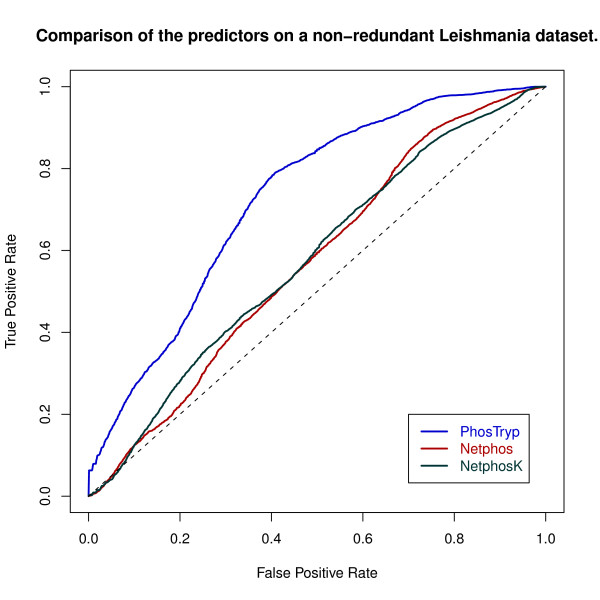
**ROC curves obtained with PhosTryp, Netphos and NetphosK on the *Leishmania *dataset**. The curves represent the average of 100 bootstrap replicates.

Figure 1 displays the Receiver operating characteristic (ROC) curves corresponding to the application of each method to the non-redundant test set. PhosTryp therefore represents a 17% performance improvement over non organism-specifc methods for the prediction of phosphorylation sites in *Leishmania*.

### PhosTryp captures phosphorylation features specific to *Leishmania*

The improved performance of PhosTryp could be explained by differences in sequence specificity between the *Leishmania *kinases and the kinases of other, better characterized, organisms. To investigate this possibility we used NetPhorest [31], a collection of 125 sequence-based classifiers that predicts which kinase group is more likely to phosphorylate a given substrate. The output of NetPhorest is a score representing the probability that a given kinase group phosphorylates the peptide under analysis. In this analysis we only considered the highest scoring kinase group for each peptide. Obviously the more the sequence of the peptide is similar to the *consensus *recognition sequence of the kinase the higher the score. The majority of the data in NetPhorest comes from experiments performed with human kinases and kinases from model organisms. Therefore the score of a peptide is a direct indication of the overlap between the specificity of the kinase responsible for its phosphorylation and the specificity of kinases from well-characterized organisms.

We divided our phosphorylation sites in two groups: the sites that were predicted correctly by PhosTryp and Netphos, and the sites that were false negatives according to Netphos and true positive for our method. The latter group, which was missed by Netphos but not by PhosTryp, could contain peptides with *Leishmania*-specific recognition sequences. Indeed the average NetPhorest score for this set of peptides is 0.24, lower than the 0.34 obtained with the peptides that were correctly predicted by our method and Netphos (p < 8.6e-16, Wilcoxon test). These results further confirm that PhosTryp, by training on *Leishmania *sequences, is able to identify phosphorylation events that are specific to this organism.

### Search for new motifs in peptides predicted by PhosTryp

One possible explanation for the increased performance of PhosTryp compared to Netphos and NetphosK is that the dataset we used contains *Leishmania*-specific phosphorylation motifs. Therefore we extracted all the peptides which were correctly predicted by PhosTryp but not by Netphos, to assess whether they contain novel phosphorylation motifs. We used the motif-x server with default parameters for motifs extraction [32] using as background dataset the whole *L. infantum *proteome. To further assess the novelty of the motifs we visually compared the sequence logos with an extensive collection of known kinase recognition logos [31].

**Figure 2:**
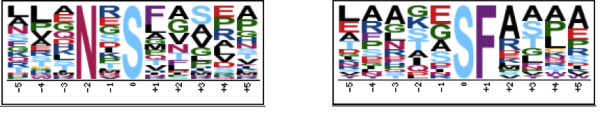
**Novel motifs identified by the motif-x webserver on the set of peptides correctly predicted by PhosTryp but not by Netphos**. The two motifs were identified in the *Leishmania infantum *dataset. The left motif has a 6.01 fold enrichment in the phosphopeptides dataset with respect to the whole *Leishmania *proteome while the right one has a 5.11 fold enrichment. All the motifs have a significance <=10e-6.

This analysis resulted in the identification of two possible *Leishmania*-specific motifs for phosphorylation on serine (see Figure 2). The first motif, Nx**S**, has a 6.01 fold enrichment in the phosphopeptides dataset with respect to the whole *Leishmania *proteome while the second one, **S**F, has a 5.11 fold enrichment. All the motifs have a significance <=10e-6. Clearly the biological significance of these motifs should be experimentally tested. However the enrichment in the phosphopeptides dataset with respect to the proteome shows that these are not simply residues over-represented by chance at proximal positions.

### Testing the predictor on other Trypanosomatids

We decided to investigate how the increase in performance with respect to NetPhos and NetPhosK translated to trypansomatids other than *Leishmania infantum*. To this end we collected two other sets of phosphorylation sites from two recent phosphoproteomics experiments performed in *Trypanosoma cruzi *[33] and *T. brucei *[6]. For each set we collected, similarly to what we did for *L. infantum*, an equal number of negative sites by a random sampling of the proteome. The *T. cruzi *dataset comprised 356 peptides (half of which positives and the other half negatives) while the *T. brucei *dataset consisted of 3056 peptides.

**Figure 3:**
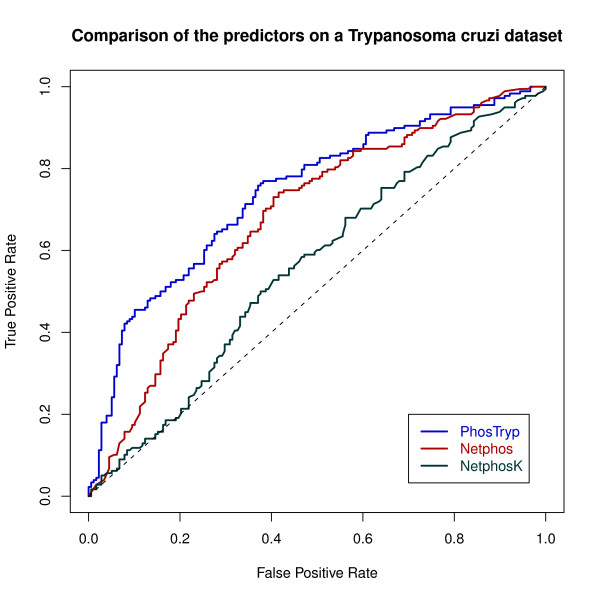
**ROC curves obtained with PhosTryp, Netphos and NetphosK on the *T. cruzi *dataset**.

**Figure 4:**
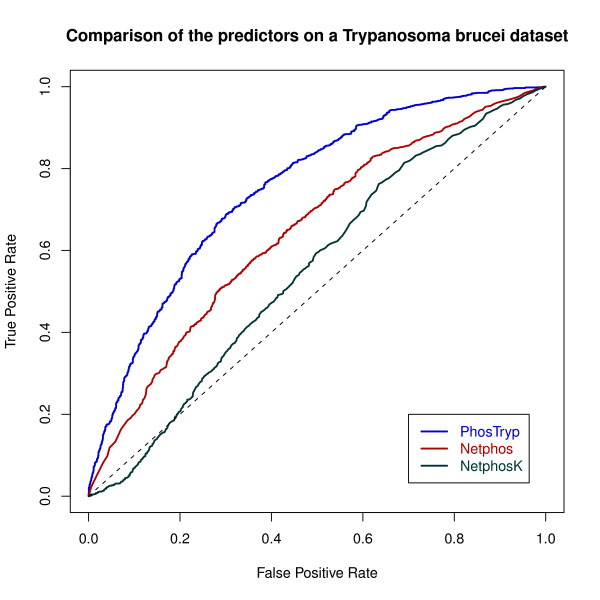
**ROC curves obtained with PhosTryp, Netphos and NetphosK on the *T. brucei *dataset**.

We then used the SVM that had the best performance on *L. infantum *to classify the peptides in the two new datasets. We obtained an AUC of 0.74 on the *T. cruzi *dataset and of 0.75 on *T. brucei *(Figures 3 and 4). Netphos had a lower performance of 0.68 and 0.65 respectively. The results with NetphosK were even worse, with an AUC of 0,56 on *T. cruzi *and 0.55 on *T. brucei *(see table 3). These results show that PhosTryp, which was trained on *Leishmania infantum*, performs better than generic predictors when applied to this group of organisms.

**Table 3:** Comparison of the results obtained with PhosTryp, Netphos and NetphosK on the *T.brucei *and *T. cruzi *datasets

Method	*T. brucei*	*T. cruzi*
PhosTryp	0,753	0,741

Netphos	0,647	0,680

NetphosK	0,553	0,560

In order to verify whether this improvement was simply due to the identification of phosphorylation sites in the orthologues of the proteins used for training, we used the orthoMCL database [34] to exclude from this test any sequence belonging to the same ortholog group as one of the training proteins. Following this step there is a 0.01 reduction in AUC on both the datasets from *T. cruzi *and *T. brucei*. We can therefore conclude that PhosTryp, after being trained on *L. infantum*, succeeded in capturing phosphorylation features that are specific to trypanosomatids.

### Development of a predictor for organisms of the family Trypanosomatidae

Our results show that a predictor trained on *Leishmania*-specific data performs better than generic predictors even when applied to the related organisms *T. cruzi *and *T. brucei*. This is an important point because it shows that it is possible to improve the prediction of phosphorylation sites in *Trypanosomatidae *using data specific to a single organism of this group. Clearly, since phosphorylation data is available for *T. cruzi *and *T. brucei *as well, the best strategy to develop a predictor specific for Trypanosomatidae is to also use these peptides in the training. We therefore developed another predictor that was trained on a combined dataset including phosphopeptides from *L infantum, T. cruzi *and *T. brucei*. As previously described for the *Leishmania *SVM we split the data into 80% training and 20% test. Moreover the peptides in the test set that had more than 50% identity with one of the peptides used during the training were removed.

On the test including the peptides from all the organisms the method has an AUC of 0.78 (see Table 4). The score threshold that maximizes the MCC is 0.49. We also evaluated the performance on the test peptides specific to each organism. The sequences from *L. infantum, T. brucei *and *T. cruzi *were predicted with an AUC of 0.75, 0.79, 0.79 respectively. As expected the performance of the method increases when more data is used for training. This predictor is the one used in the webserver available at http://phostryp.bio.uniroma2.it.

**Table 4:** Comparison of the results obtained with PhosTryp trained on all organisms, Netphos and NetphosK

Method	*L. infantum*	*T. brucei*	*T. cruzi*	All organisms
PhosTryp	0.746 ± 0.008	0.794 ± 0.005	0.788 ± 0.013	0.776 ± 0.004

Netphos	0.654 ± 0.007	0.646 ± 0.006	0.723 ± 0.014	0.659 ± 0.005

NetphosK	0.520 ± 0.010	0.585 ± 0.006	0.456 ± 0.019	0.557 ± 0.005

## Conclusions

We have described the development of PhosTryp, the first phosphorylation site predictor specific for trypanosomatids.

PhosTryp uses an SVM approach and was initially trained on an extensive collection of 1176 phosphorylation sites derived from large-scale phosphoproteomics experiments conducted in *Leishmania*. The predictor was tested on a dataset that did not contain peptides similar to those used during the training and obtained an AUC of 0.74. This result represents a 17% improvement over the results obtained with Netphos, a generic, non organism-specific, predictor.

We investigated in more detail the peptides that are correctly predicted by PhosTryp but not by Netphos. This analysis showed that these peptides have, on average, significant differences in their kinase recognition sequences when compared with phosphorylation sites from more extensively studied model organisms. Moreover we identified two possible novel serine phosphorylation motifs specific for *Leishmania*. These results show that our method performs better than generic predictors because it captures *Leishmania*-specific phosphorylation features.

We also verified that this perfomance improvement extends to other organisms in the trypanosomatids group. More specifically PhosTryp represents a 10% performance improvement over Nepthos in the prediction of *T. brucei *phosphorylation sites and a 6% improvement when applied to data from *T. cruzi*.

These results show that it is possible to improve phosphorylation site prediction in trypanosomatids using data specific to a single organism of this group. In order to maximize the performance and usefulness of PhosTryp we retrained the predictor combining the data from *L. infantum, T. cruzi *and *T. brucei*. As expected this combined predictor shows an increase in performance.

In conclusion our work highlights the usefulness of developing predictors starting from species-specific data, so as to capture features which are characteristic of a given organism, or, such as in this case, group of organisms. We have made available PhosTryp as a web server at http://phostryp.bio.uniroma2.it.

## Methods

### Positive dataset

The phosphorylation sites used in this study are derived from phosphoproteomics experiments conducted in *Leishmania donovani *using the fully annotated genome database of the closely related *L. infantum *(http://www.genedb.org) [35] (i.e. all the sequences used in this work are from *L. infantum*). A portion of these peptides has already been published [7]. The remainder was identified using the following experimental procedure (Tsigankov *et al*., in preparation).

A cloned line of *L. donovani *1SR was grown and submitted to differentiation as described in [36]. Phosphatase inhibitors were used during cell harvesting. Frozen cell pellets were lysed using a buffer that contained deoxy-cholate and phosphatase inhibitors as described in [37]. One milligram of protein from each time point was reduced with dithiothreitol and cysteine sulfhydryls alkylated with iodoacetamide, and then subjected to 20 μg of trypsin for 16 h at 37°C. The resultant peptides were mixed with TiO2 beads, and phosphopeptides were eluted in 2 steps, using 30 and 50% ACN in 0.5% NH4OH. The eluted peptides were subjected to LC-MS/MS analysis. All data files were searched for protein identification using Protein Pilot (V 2.01) and MASCOT. Data was searched against the *L. infantum *ver. 3 database.

The peptides used in this work represent the largest available reportoire of *Leishmania *phosphorylation sites. Since the dataset contained a low number of tyrosine phosphorylation sites we decided to eliminate them and only focus on serine and threonine. Our work is therefore based on 1176 phosphorylation sites, 966 on serine and 210 on threonine, mapping to 482 phosphoproteins. We obtained our positive set by extracting a window of -5/+5 residues around the phosphorylation site. The redundancy of the dataset was reduced by discarding peptides having more than 50% identity (including the phosphorylated residue) with another peptide in the set.

### Negative dataset

To construct a negative dataset we firstly extracted all the serine and threonine residues with their surrounding amino acids (-5/+5) from the *L. infantum *proteome after excluding the proteins with experimentally identified phosphorylated residues. We then performed a random sampling of these peptides in order to have negative and positive sets of the same size. The sampling process preserved the same 8:2 ratio of serines to threonines that was found in the positive dataset. As done for the positive set, the redundancy of the negative peptides was reduced using a 50% sequence identity cutoff.

### Support Vector Machine features

For each peptide, the features we included as variables in the Support Vector Machine (SVM) were: the amino acid sequence, the secondary structure and the disorder prediction for the site, and a feature dependent on the composition of a window of +/- 2 residues around the phosphorylation site. Each feature is described in more detail in the following paragraphs.

### Sequence features

The sequence was given as input to the SVM using two different representations: the standard orthogonal binary encoding, and an encoding based on the substitution values in a PAM30 matrix. More specifically each one of the 11 residues of the peptide is represented by a vector of 20 elements, corresponding to the 20 different aminoacids. When the binary encoding is used the column corresponding to the identity of the aminoacid at a specific position of the peptide has value 1, while the remaining 19 columns are 0. The alternative encoding assigns to each of the 20 columns the value for the substitution of the residue in the peptide with the aminoacid corresponding to the column.

The substitution matrix-based encoding is clearly less stringent than the orthogonal encoding. However we did not want to be excessively permissive as even a single mutation can have a profound effect on the interaction of a kinase with its substrate. Therefore we chose to use the PAM30 matrix which is fairly stringent and is also the default used by the NCBI BLAST server when dealing with peptide queries.

### Secondary structure and disorder features

The secondary structure of each residue was predicted using the PSIPRED software [38] (the whole sequence of the protein was used as input). We encoded this prediction as a binary feature according to whether the phosphorylation site is located in a coil or not. Similarly we predicted the order/disorder state of each residue using the Remark465 predictor of DisEMBL [39]. This was also coded as a binary feature according to whether the site is predicted to lie in a disordered region or not.

### Residue composition feature

The last feature we included in our predictor depends on the identity (but not position) of the residues in a window of +/- 2 aminoacids around the site. Firstly we calculated the number of occurrences of each aminoacid in the positive and negative sets, normalizing by the size of each set. We then defined a propensity value as the logarithm of the ratio between the occurrence of each aminoacid in the positive and negative sets. The propensity scores of the four residues in the +/- 2 window were then summed to obtain a final value which was given as input to the SVM.

### SVM training

We used 80% of the positive and negative sets to train the SVM. The remaining peptides were used as test. The SVM training and testing procedure was written in R, using the package e1071. We trained 4 SVMs: each one of the two sequence encodings (orthogonal and matrix-based) was tried with and without the extra, non-sequence, features (secondary structure, disorder predictions and residue composition feature). We used the Radial Basis Function as kernel for regression. This means that each classifier outputs a numeric value according to the likelihood that a residue is phosphorylated.

For each SVM, we performed a grid search to select the best values for the kernel function parameters: gamma, cost and epsilon. The grid search method we implemented is an iterative process that starts from the full range of values for each parameter. For the cost, i.e. the penalty factor, we centered the search around a value equal to the range of output values. The epsilon parameter search was restricted to a range of values that give good generalization capabilities [40]. The gamma parameter is known to be related to the number of features of the SVM, therefore a different range of gamma values was used for each SVM.

The range of each parameter is first discretized according to a certain step size. Then at each iteration the algorithm tests all the possible combinations of parameters values to identify the one yielding the best performance (i.e. lowest mean squared error). Each particular combination of parameters is evaluated using a 10-fold cross validation. At each subsequent iteration the range is halved, using the best value of each parameter as the center of the new range. If the new range contains points that fall outside of the initial range of the parameter the bounds are modified. This process is halted when the variation in lowest mean squared error between the current and previous iterations is less than a fixed value. The values of gamma, epsilon and cost that result in the best performance across all the iterations are selected for each SVM.

### SVM test

As previously stated 20% of the positive and negative sets were used to test the SVM. All the SVMs were tested using a 50% non-redundant test set. This dataset was obtained by discarding from the test set the peptides that shared a sequence identity greater than 50% with any of the peptides of the training set (including the phosphorylated residue). Furthermore the same redundancy reduction was applied within the dataset. Positive and negative peptides were treated separately throughout. The final non-redundant test set comprised 116 positive and 170 negative peptides. A bootstrap procedure was implemented to assess the variability of the performance measures on the final test. The bootstrap consisted of 100 samples (with replacement) of 80% of the final test set. The Area Under the ROC Curve was used as performance measures throughout this work.

### Training and testing using the combined dataset of *L. infantum, T. brucei and T. cruzi*

To derive the final version of PhosTryp we used our data from *L. infantum *combined with recently published data from the related organisms *T. cruzi *and *T. brucei*.

All the phosphopeptides from these three organisms were pooled in one set. Negative peptides were sampled from each proteome, maintaining the same proportion as found in the positive set. We reduced the redundancy using a 50% sequence identity cutoff similarly to what we did for the *L. infantum *SVM (see above). The same pipeline described above for the *L. infantum *dataset was applied for training (80% of the data) and testing (20%). The features we included in this predictor were the ones that resulted in the best performance for the *L. infantum *SVM, i.e. all the non-sequence features and the sequence in PAM30 encoding.

Two tests were performed. In one case all the sequences were kept together. In the second test we divided the test sequences according to the organism which they belong to, and we assessed the performance separately for each organism.

## Competing interests

The authors declare that they have no competing interests.

## Authors' contributions

PFG designed the study. AP analysed the data and developed the software. PT carried out the phosphoproteomics experiments. GA, GFS, DZ and MHC supervised the work. PFG and AP wrote the paper. All authors read and approved the final manuscript.
